# Spatial distributions of salt-based ions, a case study from the Hunshandake Sandy Land, China

**DOI:** 10.1371/journal.pone.0271562

**Published:** 2022-08-05

**Authors:** Zhanhong Li, Yunhu Xie, Xiaoli Ning, Xuefeng Zhang, Quansheng Hai

**Affiliations:** College of Resources and Environment, Baotou Teachers’ College, Baotou, China; Feroze Gandhi Degree College, INDIA

## Abstract

Soil water soluble base ion salt-based ion concentrations are critical parameters for estimating soil buffer capacity and vegetation productivity. Ionic content clearly covaries with the distribution of plant communities. Previous studies on salt-based ions in soils focused primarily on ion migration and its relationships with vegetation growth. Few studies have sought to characterize larger scale spatial distribution of salt-based ions or correlation with climatic and plant community characteristics. This study used ion chromatography to analyze the salt-based ion content (Ca^2+^, Mg^2+^, Na^+^ and K^+^) of surface soils from the Hunshandake sandy lands. Statistical methods were used interpret spatial variation. Results showed that the average content of salt-based ions in Hunshandake sandy land was 86.57 mg/kg. Average values ranked as Ca^2+^ > Na^+^ > K^+^ > Mg^2+^ but concentrations also exhibited uneven spatial distributions. Horizontal spatial variation in Ca^2+^, Mg^2+^ and Na^+^ ions showed these ions gradually decrease from northwest to southeast. Potassium ions (K^+^) showed no obvious spatial variation trends. Ions varied significantly across different soil layers but their average concentrations ranked as K^+^>Na^+^>Ca^2+^>Mg^2+^ (from shallow to deep). The 20–30 cm soil layer contained the highest salt ion concentrations. Of the four base ions, only K^+^ ions appeared in surface samples. In terms of water soluble base ion available salt-based ions, Ca^2+^ occurred in the highest concentrations along the north and west side of the study area. K^+^ ions occurred in the highest concentrations along the south and east sides of the study area. Na^+^ concentrations did not show a consistent spatial pattern. Statistical analysis detected significant correlations of normalized ion concentration parameters (Ca^2+^/CECT, K^+^/CEC, effective water soluble base ion salt-based ions) and the total species number, average species number and total biomass of the plant communities (P <0.05). This study can help inform understanding of soil water transport in sandy areas and provide a reference for interpreting ecosystems in arid regions.

## Introduction

Vegetation depends on soil. The physical and chemical properties of soil control the spatial distribution of herbaceous communities [1, 2]. They also determine soil quality [3]. Soil water soluble base ion salt-based ions (Ca^2+^, Mg^2+^, K^+^, Na^+^) can reduce soil water-holding capacity, soil moisture and residual moisture content of saturated air intake [4]. Loss of soil water soluble base ion base ions influences soil buffer capacity and vegetation productivity. Effective grassland ecosystem management depends on mitigating these effects [5]. Numerous studies have found that among other environmental factors, groundwater level and soil based ion content are primary factors influencing the characteristics and distribution of plant communities found in local depressions or at the foot of hillslopes [6–8]. The movement and evaporation of water in soil can cause changes in soil salinity. Water movement in soil depends on groundwater depth [9, 10]. In arid and semi-arid areas, soil salt-based ions migrate within soil by groundwater transport [11]. In areas where phreatic water evaporates rapidly, soil salt content depends on groundwater salinity and soil depth [12]. Previous studies on base ions in soil show that ions play a key role in moderating soil acidification and regulating soil fertility. Base ions accumulate in the surface layer of soil. Group I and II metals form strong alkali-weak acid salts which become hydrolyzed to produce OH^-^ and soil alkalization. These processes impact soil quality. Wind erosion [13], human disturbance [14] and grazing intensity can also influence salt-based ions in soil [3].

Hunshandake sandy land is a major desert area in China. Overgrazing and drought conditions caused by climate change and reclamation have led to degradation of grassland which, in turn, has led to erosion and sediment mobilization. Previous studies of Hunshandake sandy land have focused primarily on plant communities, desertification, degradation and mitigation / control efforts. The distribution of soil base ions can influence groundwater and soil moisture dynamics. Feedback loops associated with these processes contribute to regional environmental change. Taking a regional scale perspective, This study describes concentrations and spatial distribution characteristics of soil base ions. Variation in soil base ions highlight relationships, migration and interdependencies with climatic factors and plant communities. A systematic understanding of soil water and ion dynamics can advance understanding for similar regions and guide management and protection of these ecologically fragile ecosystems. Hunshandake sandy land desertification governance research results of inestimable significance in the world, the study on Hunshandake sandy land as the research area, develop base ion spatial distribution characteristics of research, On the one hand, it makes up for the gap that previous studies are only limited to desertification control or macro scale. On the other hand, it can lay a foundation for micro research on Hunshandake sandy land after ecological restoration. It is of great significance for soil improvement and sustainable utilization.

### Site description

Hunshandake sandy land is located in the middle of the Xilinguole Grassland of Inner Mongolia (E115°34′35″, N42°57′23″). It extends 260 km in length from east to west and spans 50–100 km in width from north to south. Western areas meet criteria for an arid climate zone while areas on its southeastern edge categorize as semi-arid but transition into desert grassland, meadow grassland and typical grassland. Temperature and precipitation conditions for this area categorize as a temperate continental monsoon climate. The average annual temperature of 1.75–3.23 °C and average annual precipitation of 264.6–368.7 mm decrease from southeast to northwest. Heat and evaporation increase from southeast to northwest. The region experiences strong winds and 50–70 annual wind days. Sandy soil is mainly wind-sand soil developed on sandy parent material with limited growth of vegetation, low organic matter content and poor stability. Meadow soil and swamp meadow soil are often distributed in a steppe belt of hilly lowland and sandy land. Most of these areas have become salinized to varying degrees. The sand belt is surrounded by typical grassland chestnut soil, with aeolian sandy soil, brown calcium soil, meadow soil and other soil types.

## Materials and methods

### Soil sample collection

**Fig 1:**
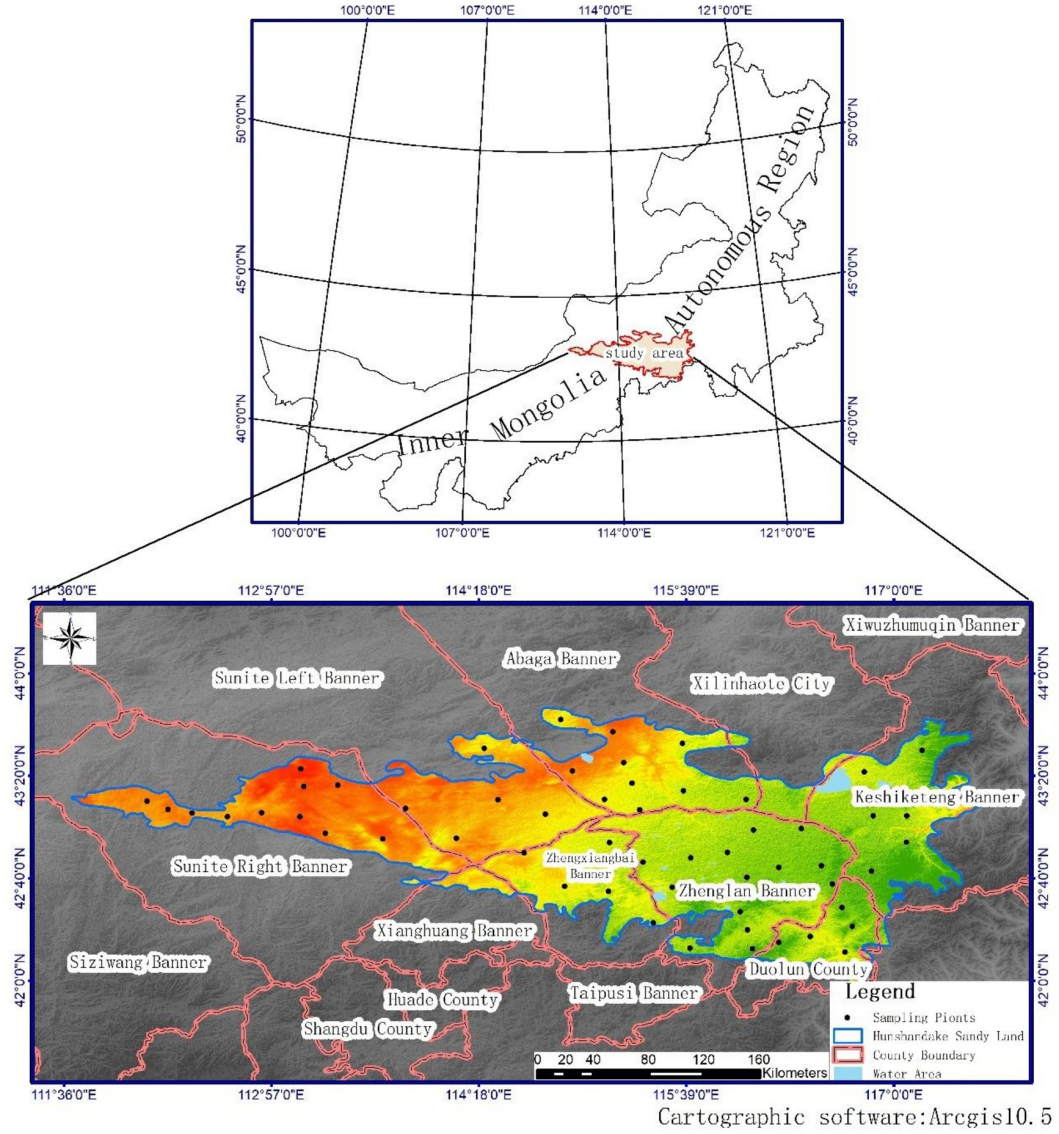
Location of study area and map of sampling points throughout Hunshandake sandy land.

**Fig 2:**
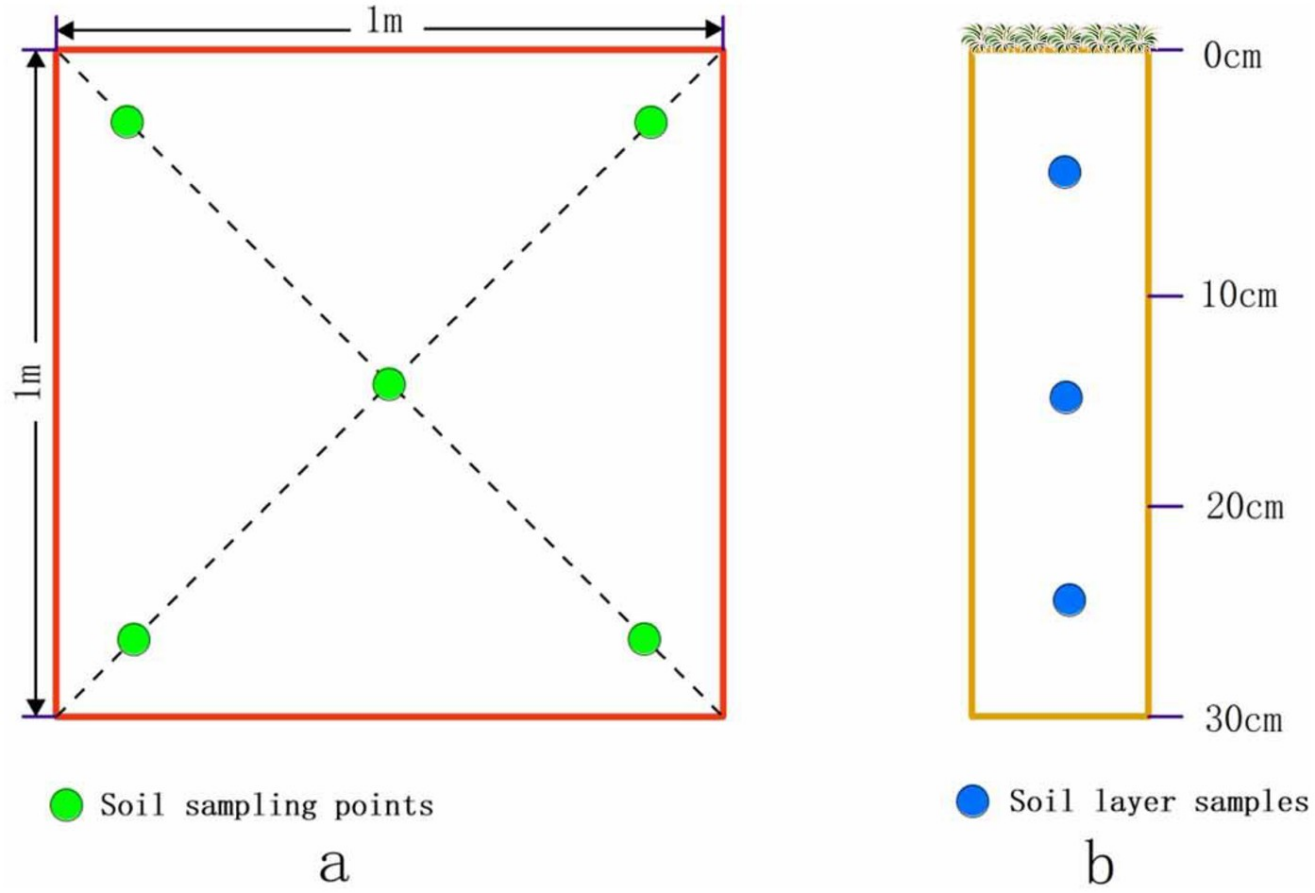
Schematic diagram of soil sampling (a: Layout of sample points; b: Sampling of soil profile).

Vegetation and soil surveys were carried out in Hunshandake sandy land in July 2018. Soil profiles were sampled from the locations shown in Fig 1 (black dots). The sampling strategy used three sampling points randomly selected from within a set of 1 x 1 m sample plots. Sample plots were divided into five soil sampling points according to a diagonal layout (Fig 2a). Each soil sampling point included three soil layers (0–10 cm, 10–20 cm and 20–30 cm depth) (Fig 2b). Soil samples were transported back to the laboratory for air drying, removal of impurities and further sample processing.

### Determination of total base ions in soil by ion chromatography

The total water soluble base ion salt-based ions can serve as an index for soil adsorption performance. This information also constraints soil formation and properties. The pretreated soil samples were dried and passed through a 1 mm soil sieve. The ion extraction used a 1:5 ratio of ultra-pure water to soil. The slurry was agitated for 3 minutes at a frequency of 300 R/min. After centrifuge separation, the supernatant was filtered and the ion content was determined by ion chromatography (ics-600) [15].

### Spatial difference analysis using the Inverse Distance Weighting (IDW) multivariate interpolation method

The Inverse Distance Weighting (IDW) Multivariate Interpolation Method uses principles of geographic similarity. The distance between interpolation points and sample points is treated as a weighting and used to calculate a weighted average. The closer the sample point to the interpolation point, the greater the weight. The formula for analyzing the spatial distribution of base ions is as follows:

$$\hat{z}\left(s_{0}\right)=\frac{\sum_{i=1}^{n}d_{i}^{-p}z_{\left(s_{i}\right)}}{\sum_{i=1}^{n}d_{i}^{-p}}$$ (1)

Where $\hat{z}\left(s_{0}\right)$ is the predicted value at *S*_0_, $z_{\left(s_{i}\right)}$ is the measured value obtained at *s*_*i*_, n is the number of sample points around the prediction points used in the budget process, *d*_*i*_ is the distance between prediction point *S*_0_ and each known sampling point *s*_*i*_ and p is the index value.

The research content of this project is carried out under the premise that the ecological environment is not destroyed, and does not need the permission of relevant departments.

## Results and analysis

### Total content and spatial distribution of salt-based ions

Analysis of samples from 0–30 cm depth for each sample point found that the total salt-based ions in the study area ranged from 481.1 cmol/kg to 3.3 cmol/kg with an average of 86.57 mg/kg and a standard deviation of 95.42. Different sample points showed considerable dispersion in salt-based ion content. Among them, Ca^2+^ content ranged from 0–235.3 mg/kg with an average value of 47.78 mg/kg and a standard deviation of 58.04. K^+^ ranged from 0–32.1 mg/kg with an average value of 10.57 mg/kg and a standard deviation of 13.6. Mg^2+^ ranged from 0–24.7 mg/kg with an average value of 4.26 mg/kg and a standard deviation of 6.59. Na^+^ ion content ranged from 0–201.9 mg/kg with an average value of 23.96 mg/kg and standard deviation of 43.15. The average values for each base ion ranked as Ca^2+^> Na^+^> K^+^> Mg^2+^.

**Fig 3:**
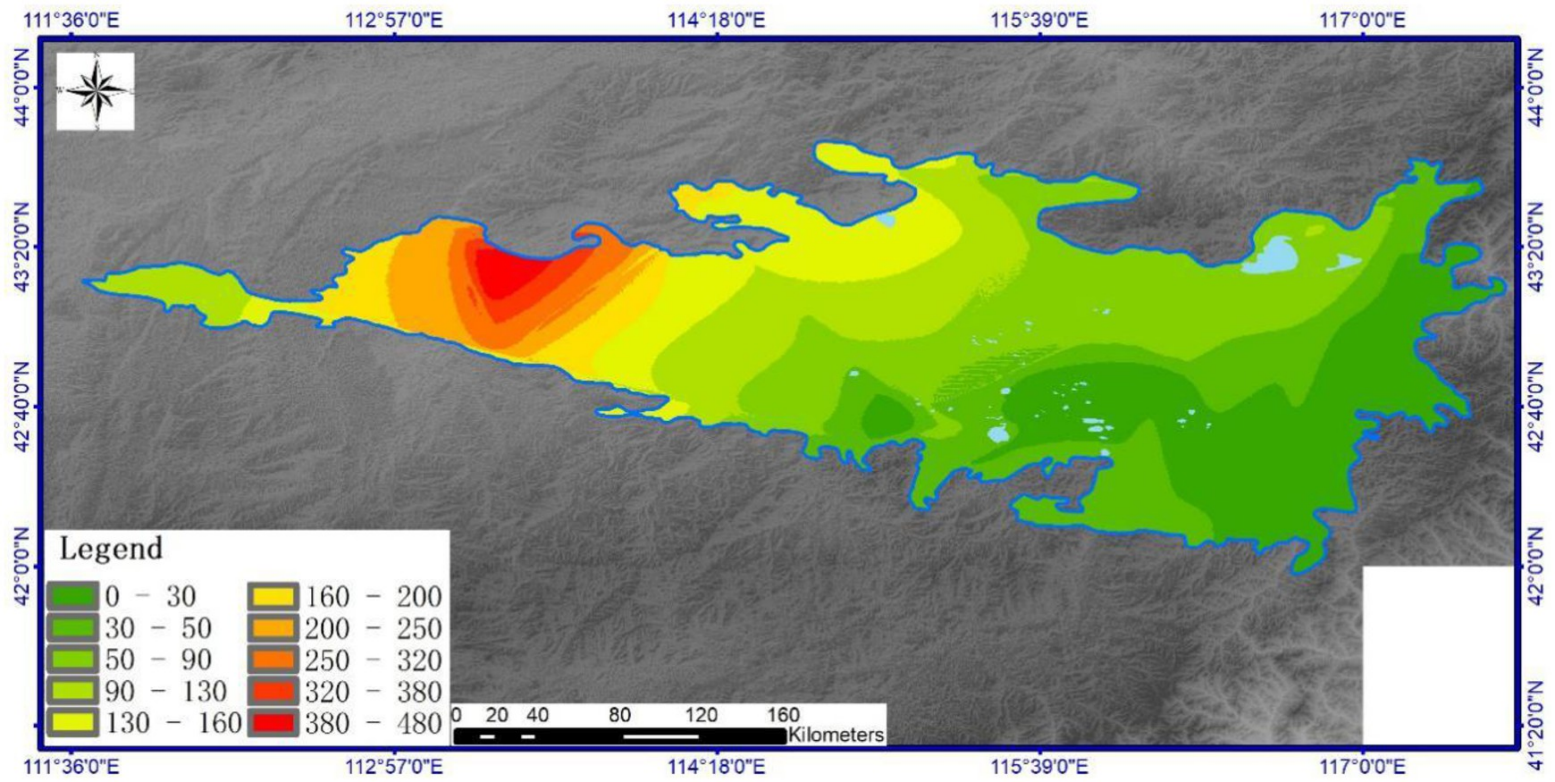
Spatial distribution of total base ions (mg/kg) in soils across Hunshandake sandy land(Cartographic software:Arcgis10.5).

Spatial analysis of total base ion content (Fig 3) shows that the highest total salt-based ion concentrations occur in central and western zones of the study area where values reach 480 mg/kg. Total salt-based ion content exceeds 160 cmol/kg in 12.8% of the total study area. Total salt-based ions content falls below 100 cmol/kg in 53% of the total study area. Similar to spatial trends in regional temperature and evaporation, ion content gradually increases from southeast to northwest. The similar distributions between precipitation and salt-based ions in soil indicates a mechanistic relationship and demonstrates that the approach carries implications for regional hydrology.

### Horizontal spatial distribution of different salt-based ions

**Fig 4:**
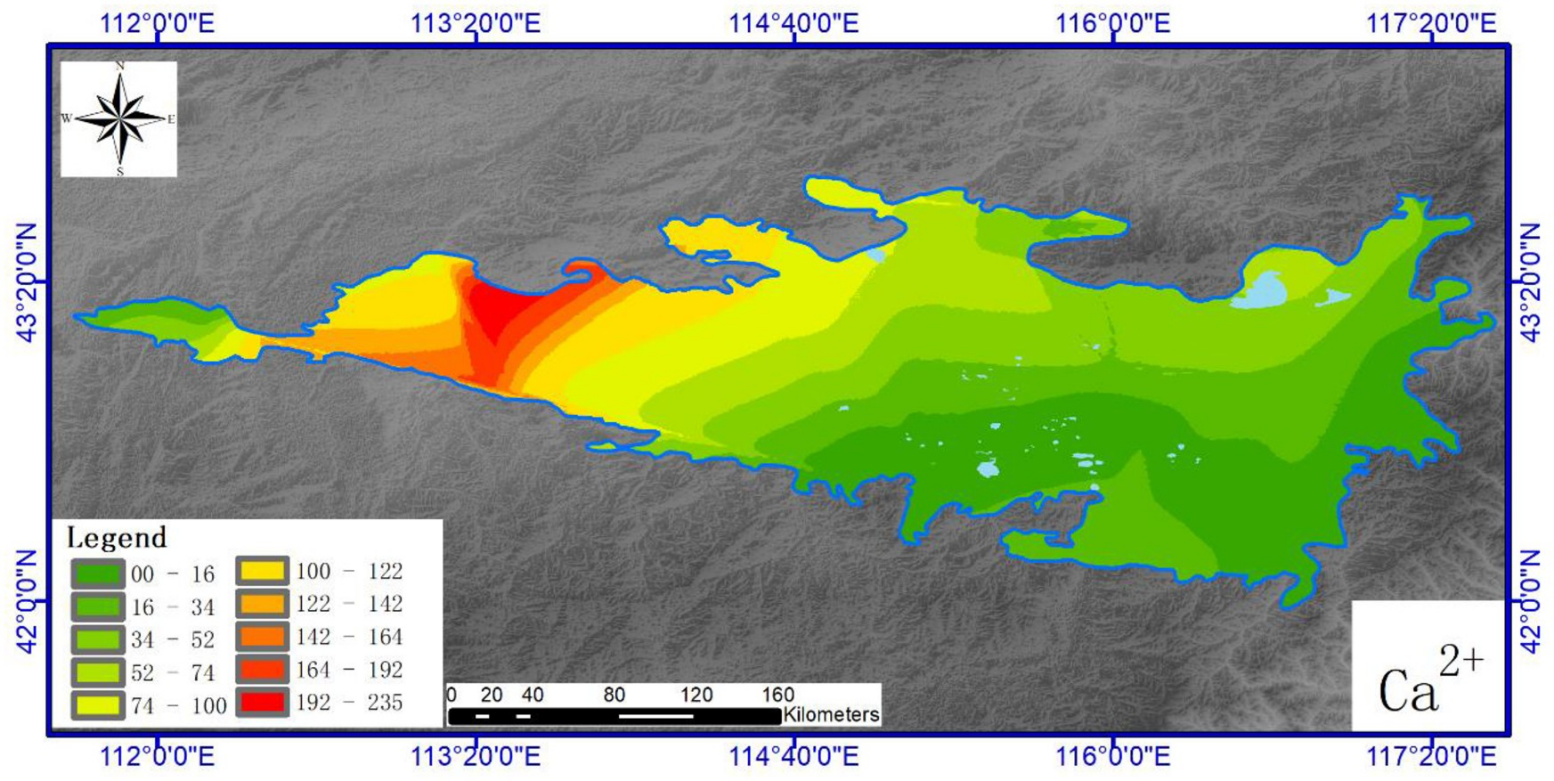
Spatial distribution of soil Ca^2+^ in Hunshandake sandy land (Cartographic software:Arcgis10.5).

**Fig 5:**
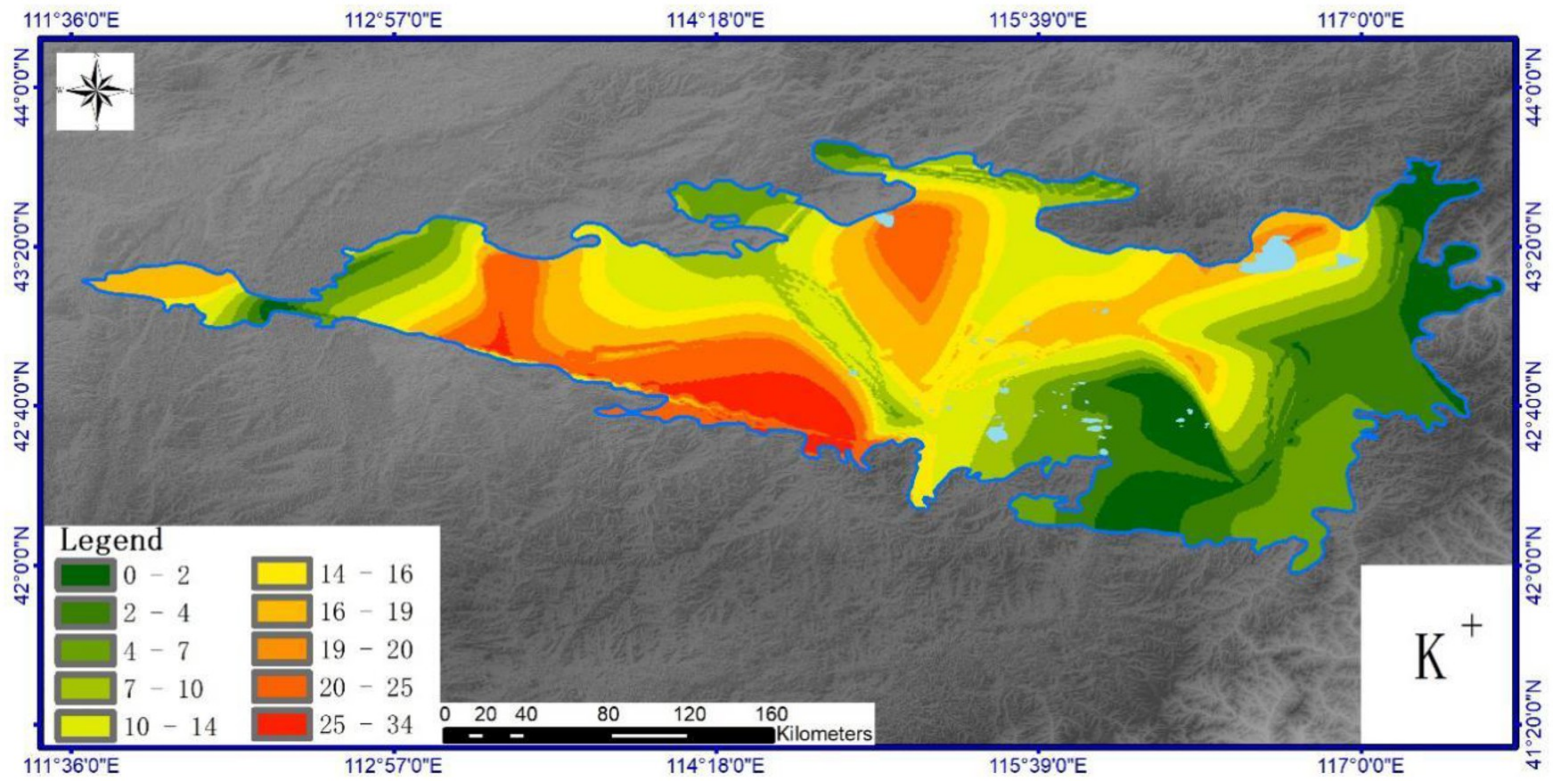
Spatial distribution of soil K^+^ in Hunshandake sandy land (Cartographic software:Arcgis10.5).

**Fig 6:**
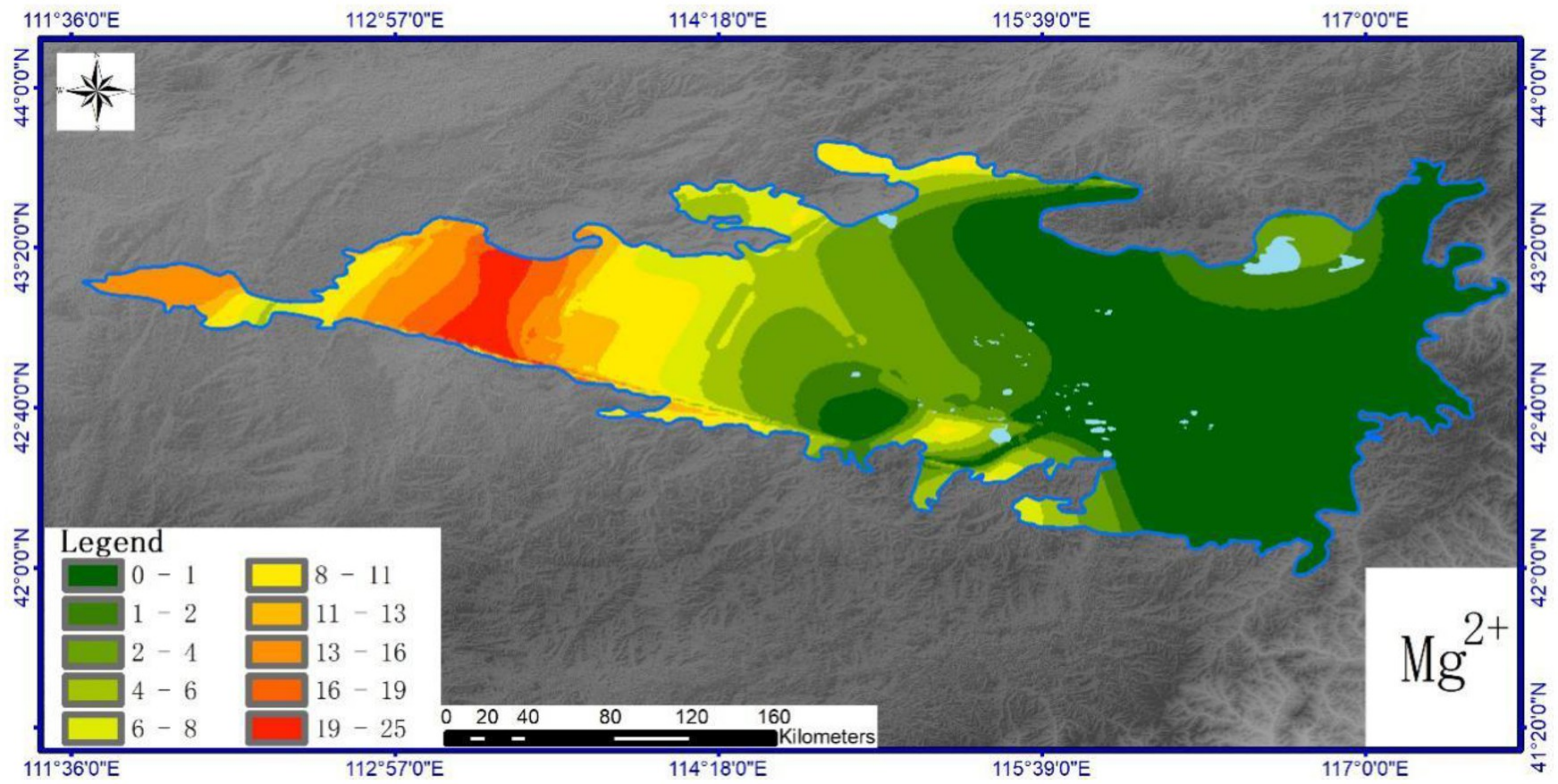
Spatial distribution of soil Mg^2+^ in Hunshandake sandy land (Cartographic software:Arcgis10.5).

**Fig 7:**
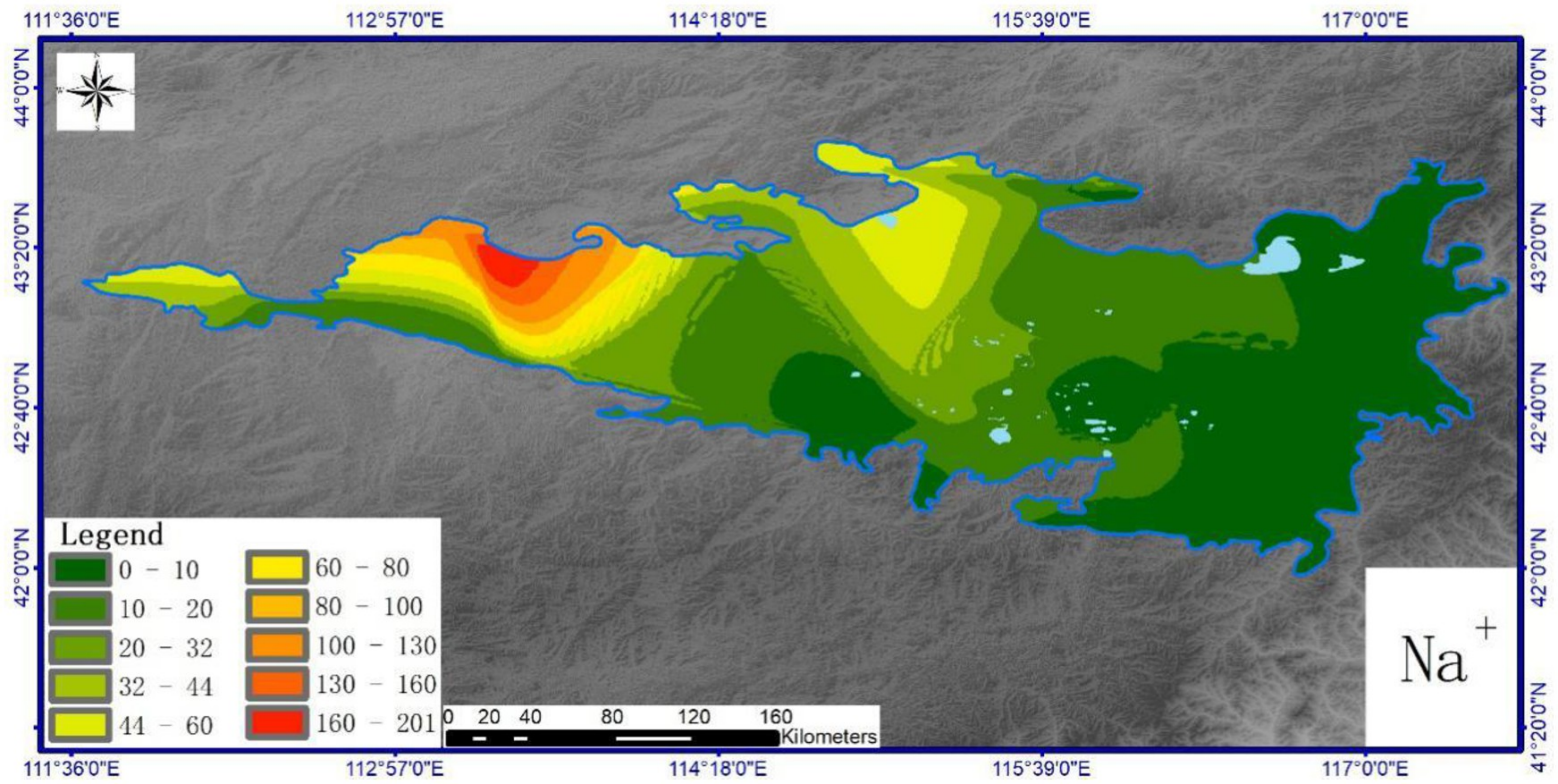
Spatial distribution of soil Na^+^ in Hunshandake sandy land (Cartographic software:Arcgis10.5).

Figs 4–7 show the horizontal spatial distribution for different individual ion parameters. Ca^2+^, Mg^2+^ and Na^+^ showed similar horizontal spatial distributions with concentrations gradually decreasing from northwest to east and south. K^+^ concentrations did not adhere to this general spatial pattern. All four ion types showed greater variation in the west than in the north. Overall concentrations for each ion in the western region exceeded those measured from the eastern region. The western region also exhibits higher precipitation rates and vegetation coverage than the eastern part of the study area.

### Vertical distribution of salt-based ions

**Fig 8:**
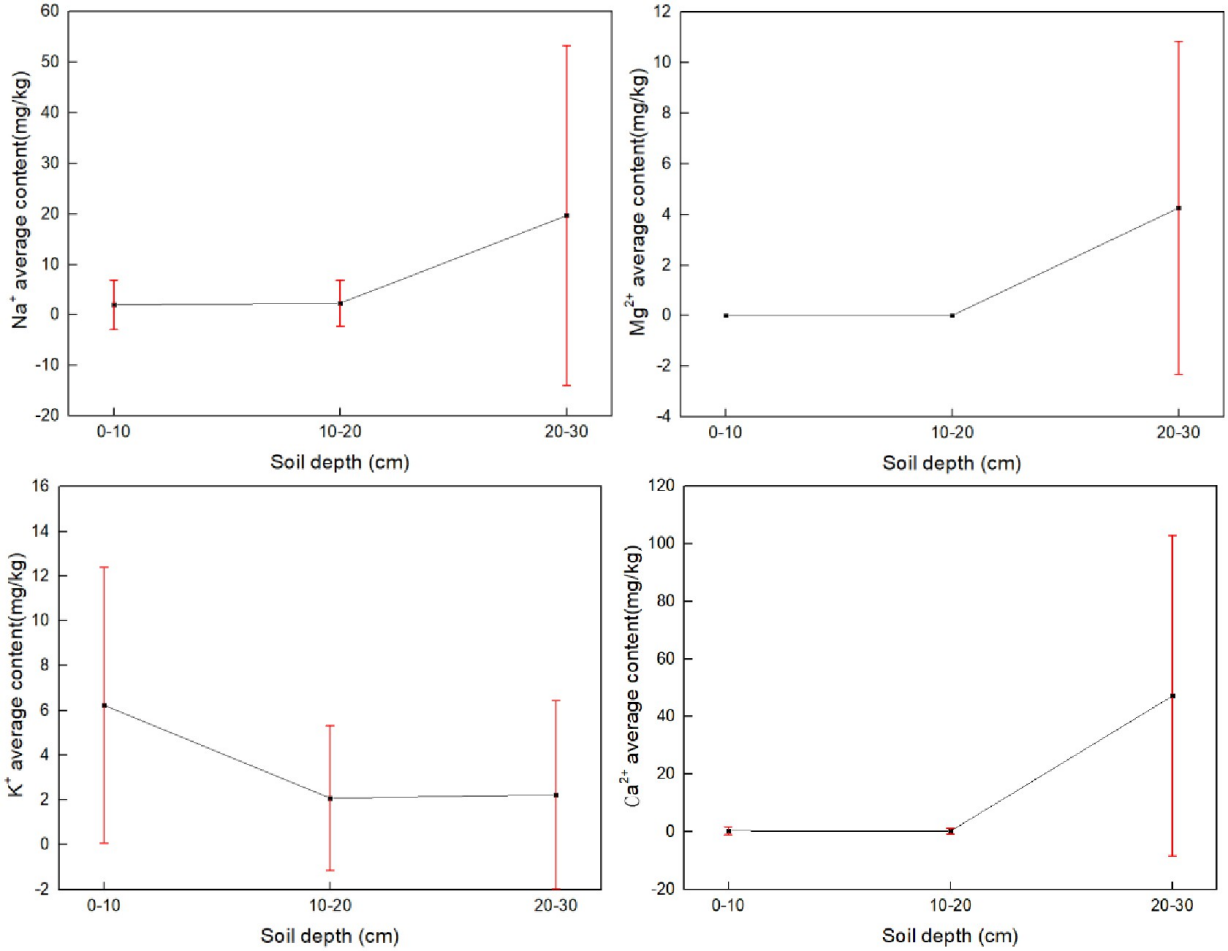
Base ion concentrations for different soil depths (Cartographic software:Origin2008).

The four base ions exhibited considerable vertical variation in soil profiles collected throughout the study area (Fig 8). Na^+^, Mg^2+^ and Ca^2+^ especially showed consistent distributions. Average Na^+^ and Ca^2+^ values for 0–10 cm depth were 1.98 mg/kg and 0.34 mg/kg, respectively. Average Na^+^ and Ca^2+^ values for 10–20 cm soil depth were 2.29 mg/kg and 0 mg/kg, respectively. This gives respective increases of 15.67% and -41.18% for Na+ and Ca^2^+ relative to their 0–10 cm soil depth values. Na^+^ content thus increased while Ca^2+^ content decreased. Meanwhile, no Mg^2+^ was detected in 0–20 cm soil layers. Na^+^ and Ca^2+^ concentrations increased significantly at 20–30 cm depth (to 19.68 and 47.24 mg/kg, respectively). Mg^2+^ increased to a value of 4.26 mg/kg in the 20–30 cm interval. K+ ions showed decreasing values with depth. Concentrations at 0–10 cm accounted for 41.37% of the total K+ ions in the profile range. This indicates significant surface accumulation of K+. The general trends described above relate to and accord with the character of the regional environment. Low silt and clay content in the soil lead to poor water holding capacity and strong permeability. Capillary water cannot reach the surface. This reduces water evaporation and lessens the accumulation of surface salt. Thus the soil does not show significant salinization. Precipitation also influences salt concentrations in the soil. As precipitation increases, water transports salts in solution thereby leaching base ions to greater depths. The Hunshandake sandy land receives between 200–400 mm annual precipitation. Precipitation increases gradually from west to east, so ion concentration depths increase to the east of Otindag sandy land. Ca^2+^ and Mg^2+^ occurred in very low concentrations at 0–10 cm and 10–20 cm soil depth. Mg^2+^ fell below detection limits indicating that the regional water capillary transpiration does not suffice to transport high valence ions. This may reflect a very deep groundwater level or may arise from the high sand content of the regional soil and its associated ability to form capillary pores. The Na^+^ and K^+^ content in the 0–10 cm surface layer significantly exceeded that observed for the divalent ions. Vertical average values for ions ranked as follows K^+^> Na^+^>Ca^2+^>Mg^2+^.

### Spatial distributions for effective water soluble base ion base ions

Ca^2+^, Mg^2+^ and K^+^ are essential nutrients in terrestrial ecosystems and represent the main components for soil water soluble base ion base ions. The exchange capacity of these base ions and their interaction with nitrogen, phosphorus and other nutrients are important soil chemical processes that contribute to biogeochemical cycles [16, 17]. The total amount of water soluble base ion base ions helps constrain the efficiency of ion exchange processes.

Samples from different sites showed variation in the proportions of the main water soluble base ion base ions. Samples exhibited consistently low proportions of Mg^2+^ from locations that represented 3.52% of the total sample points. Mg^2+^ was effectively absent from 50% of sample points. Ca^2+^ exceeded a threshold of >42.8% for 39.4% of samples. In northerly areas, Na^+^ exceeded the 42.8% threshold for 36.3% of samples. However, these samples were dispersed and showed no consistent spatial pattern. K^+^ concentrations exceeded the 42.8% threshold for 24.2% of the samples. These samples mostly occurred in a southerly area where vegetation coverage is relatively high. Effective water soluble base ion base ions showed clear and consistent spatial patterns related to regional climate and vegetation. Regional precipitation gradually decreases from southeast to northwest. Northwest areas of the study area also suffer greater degradation of vegetation cover whereas vegetation is relatively good in the southeast. Spatial analysis found that for the area of lower precipitation and degraded vegetation, Ca^2+^ dominates the effective water soluble base ion base ions. Na^+^ appears to predominate water soluble base ion base ions in low-lying areas. In areas experiencing higher precipitation and relatively good vegetation cover, K^+^ predominates over Na^+^ as the most water soluble base ion base ion in low-lying areas.

**Fig 9:**
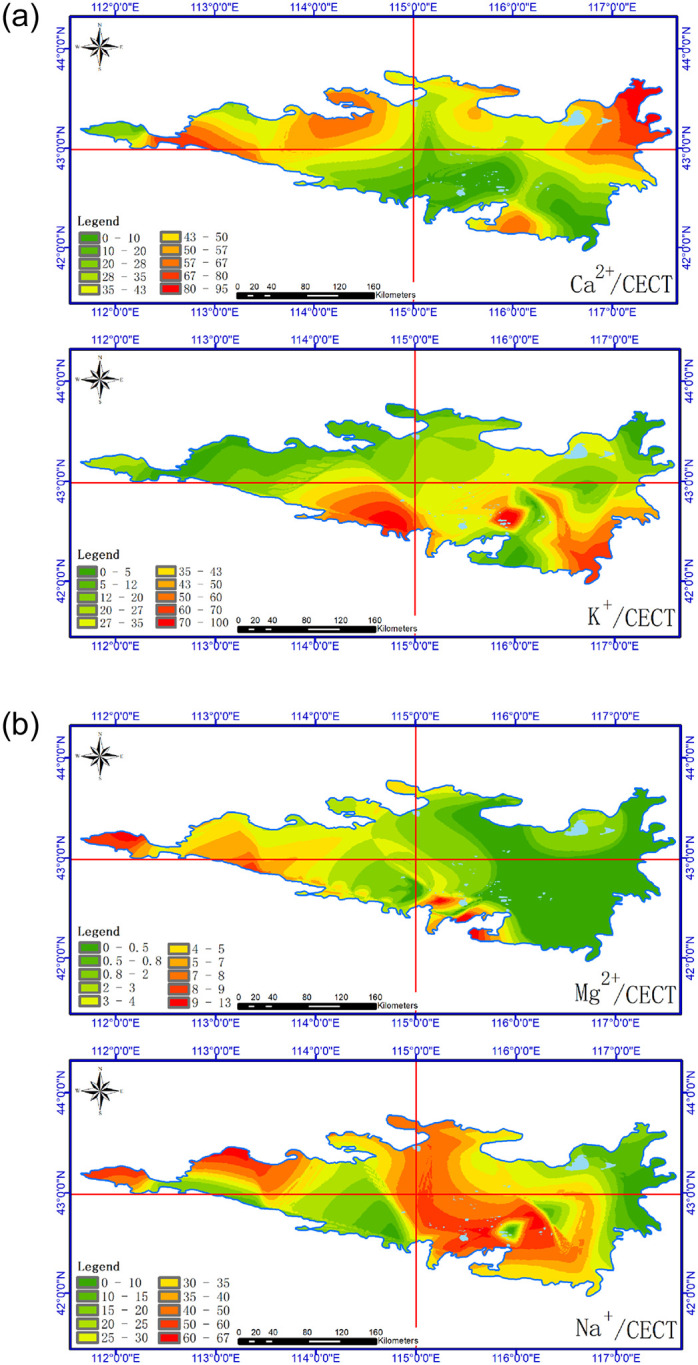
Spatial distribution characteristics of effective exchangeable base ions in soil. (Cartographic software:Arcgis10.5).

In order to further analyze spatial patterns in soil ion concentrations, the sampling area was divided into east-west and north-south quadrants along axes of 115 °E and 43 °N. As shown in Fig 9, areas with elevated levels of Ca^2+^ ions in soil occur mostly in the north and west quadrants. Samples with elevated K^+^ concentrations occur in the south and east quadrants. Samples having elevated Na^+^ concentrations do not show consistent spatial patterns. Among average values of effective water soluble base ion base ions from all samples, Na^+^ and Ca^2+^ account for similar proportions of the average values from all samples. Both of these ions occur at relatively high concentrations in specific areas.

### Correlation between base ions and plant community properties

Statistical analysis queried covariation of biomass, plant species number and species diversity with different base ions, total base ions and water soluble base ion effective base ions. Plant community parameters clearly correlated with water soluble base ion base ions but not with total or each individual base ion. The results indicate that plant communities do not rely on water soluble base ion base ions entering nutrient cycles.

According to Table 1, among water soluble base ion effective base ions, water soluble base ion Ca^2+^/CECT correlates with total biomass, total species number and average species number at the p = 0.05 level. K^+^/CECT correlates with total biomass at the p = 0.01 level and with average species number and total species number at the p = 0.05 level. Mg^2+^/CECT and Na^+^/CECT show no obvious correlation with plant community parameters. The relatively low Mg^2+^/CECT values throughout the region (with Mg^2+^ content below detection limits for most samples) offers no variation for comparison with other parameters. The absence of correlations between Na^+^/CECT and plant community parameters comports with the fact that Na^+^ does not represent an essential nutrient in plant growth.

**Table 1:** Correlation analysis between effective water soluble base ion base ions and plant community parameters.

	Water soluble base ion Ca^2+^/CECT	Water soluble base ion K^+^/CECT	Water soluble base ion Mg^2+^/CECT	Water soluble base ion Na^+^/CECT	Average biomass	Average number of species	Average diversity	Total biomass	Total species
Water soluble base ion Ca^2+^/CECT	1								
Water soluble base ion K^+^/CECT	-.713**	1							
Water soluble base ion Mg^2+^/CECT	0.047	-0.231	1						
Water soluble base ion Na^+^/CECT	-.376*	-.371*	0.073	1					
Average biomass	-0.167	0.115	-0.219	0.106	1				
Average number of species	-.355*	.410*	-0.079	-0.07	-0.268	1			
Average diversity	-0.147	0.213	-0.142	-0.069	-.396*	.772**	1		
Total biomass	-.431*	.538**	-0.045	-0.15	.623**	.401*	0.177	1	
Total species	-.348*	.417*	0.112	-0.125	-0.134	.881**	.751**	.600**	1

** Test-retest correlation significant at p<0.01.

* Test-retest correlation significant at p<0.05.

## Discussion

**Fig 10:**
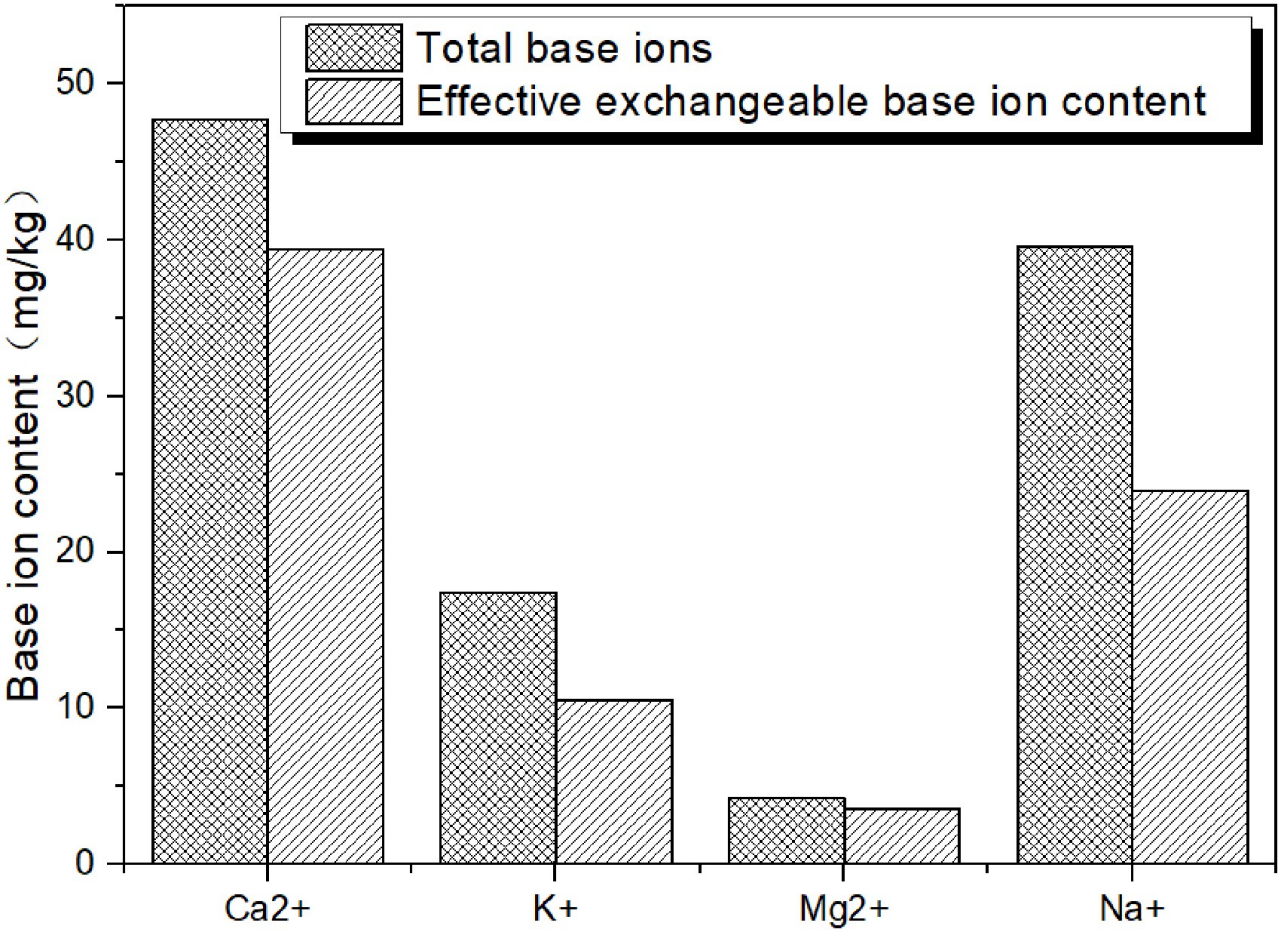
Base ions content in soil. (Cartographic software:Origin 9.1).

Variation in soil salt-based ions can arise from weathering (solution) and substitution of base ions, transport, uptake, release and other processes. Mineral weathering and dissolution generate salt ions especially thorough mineral decomposition. This weathering produces colloids (silt, clay), organic matter and combined forms (aggregates) of the two in the soil. These substrates in turn foster chemical substitution and buffering [18]. The quantity and adsorption of weathering products will therefore affect the distribution of base ions in soil [19]. Transport of base ions depends on soil moisture and groundwater. Precipitation, evaporation, irrigation, terrain differentiation, groundwater depth, soil depth, profile structure, pores and other hydrological factors influence transport and attendant distribution of base ions [20, 21]. Different plant types may absorb (or leave) base ions in the soil. Selective concentration or uptake occurs at root sites but may also depend on transpiration efficiency. Systematic, spatio-temporal patterns in plant communities can therefore impart patterns in soil salt content [22]. According to the overall analysis of the content of base ions in Hunshandake Sandy Land (Fig 10), the content of effective water soluble base ion Ca2+ base ions and effective water soluble base ion Mg2+ base ions are 39.45mg/kg and 3.52mg/kg respectively, accounting for 82.6% of the total Ca2+ ions and Mg2+ ions, respectively. The content of effective water soluble base ion K+ base ions and effective water soluble base ion Na+ base ions accounted for 60.5% of total K+ ion and 60.6% of total Na+ ion, respectively. Therefore, the content of effective water soluble base ion base ions that can be dissolved in water in Hunshandake Sandy Land is higher than 60% of the total ions, which can increase the absorption of ions by plants and promote the growth of vegetation. The study area described here experiences varying degrees of desertification which generates a layer of sand in the surface horizon. Relatively coarse-grained soil particles limit water transport among mineral particles, organic components and capillary spaces. This may cause the relatively low concentrations of Ca^2+^, Mg^2+^ and Na^+^ observed in soil surface layers. Given leeward, topographic water replenishment, good vegetation and other factors, wind erosion does not significantly impact soil. High organic matter content and mechanical properties of relative stickiness can also influence soil salinization. Plant absorption of K^+^ ions at their roots as nutrients may leave soils enriched in Na^+^. Future research should address how regional soil wind erosion, vegetation degradation and nutrient uptake influence K^+^ accumulation.

## Conclusions

The Hunshandake sandy land soils analyzed exhibited highly variable but systematic distributions in soil cations. Statistical analysis of spatial climatic, ecological and ion concentration data found correlations between water soluble base ion effective base ions and vegetation parameters. The total amount of base ions in the study area ranged from 481.1–3.3 mg/kg with an average of 86.57 mg/kg. Ca^2+^ and Na^+^ occurred in the highest concentrations throughout the study area. Average soil ion concentrations ranked as Ca^2+^>Na^+^>K^+^>Mg^2+^. In terms of their spatial distributions, Ca^2+^, Mg^2+^ and Na^+^ ions showed systematic horizontal spatial variation. Concentrations gradually decreased from the northwest to southeast zones of the study area. K^+^ concentrations did not show systematic spatial variation. Average concentrations for different ions varied with depth. The 20–30 cm soil layer contained the highest ion concentrations. Ca^2+^, Mg^2+^ and Na^+^ were not detected in surface samples whereas K^+^ ions appeared to accumulate at the surface. From shallower to deeper soil layers, ion concentrations ranked as K^+^>Na^+^>Ca^2+^>Mg^2+^. In terms of water solublebase ion effective base ions, soils containing high Ca^2+^ concentrations occurred in northerly and westerly areas. Samples exhibiting high K^+^ concentrations occurred in southerly and easterly areas. Na^+^ ions showed no systemic spatial variation. The ratios Ca^2+^/CECT and K^+^/CECT showed strong correlations with vegetation parameters including total species number, average species number and total biomass.
